# Incidence of bladder cancer in Benghazi, Libya over the past three decades

**DOI:** 10.1038/s41598-018-29187-y

**Published:** 2018-07-17

**Authors:** Narjes Saheb Sharif-Askari, Riyad Bendardaf, Fatemeh Saheb Sharif-Askari, Abdalla M. El Tabbal, Mohamed A. El Ayan

**Affiliations:** 1 Sharjah Institute for Medical Research, College of Medicine, University of Sharjah, Sharjah, United Arab Emirates; 2 Oncology unit, University Hospital Sharjah, Sharjah, United Arab Emirates; 3 Department of Clinical Sciences, College of Medicine, University of Sharjah, Sharjah, United Arab Emirates; 4 Department of Urology, Hawari Hospital, Benghazi, Libya

## Abstract

There are limited data on the disease of bladder cancer in Libya. The objective of this study was to assess the pattern of incidence and clinical presentation of bladder cancer in Benghazi, Libya. This study was a retrospective cohort analysis conducted among consecutive individuals who were diagnosed and/or were treated for bladder cancer from January 1st 1983 to December 31st 2009. A total of 835 cases of bladder tumour were recorded. The world age-standardized incidence rate was 13.1 and 1.9 per 100,000 for males and females, respectively. The mean (standard deviation) age of all patients was 63.7 (14.7). The majority of patients (n = 750, 89.8%) were male, two-thirds of which (n = 594, 79.2%) were smokers while all female patients were non-smokers. Hematuria was the most frequent presenting symptom. Most tumours were well differentiated, and transitional cell carcinoma was the most frequent histological type. The incidence of bladder cancer in Libya is lower than most developed countries, increases by aging, and is more prevalent among males. The incidence of this disease is expected to grow in developing countries such as Libya because of increase in smoking popularity, the shift to sedentary life, diabetes mellitus, and obesity.

## Introduction

Bladder cancer is the ninth most common cancer globally with 430,000 new cases reported in 2012^1^. The incidence and mortality of bladder cancer is highest among developed nations and some African countries^2^, and the incidence is lowest in Asia, Latin America and the Caribbean^1^. This geographical variation in the incidence of bladder cancer could be explained by the global distribution of risk factors which are mainly related to a region’s industrialization level such as exposure to tobacco smoke^3,4^. In North Africa, while a reduction of Schistosoma haematobium infection has resulted in a decrease in squamous cell bladder carcinoma cases^5^, it is expected that simultaneous increase in industrialization and risk factors such smoking^6^ to increase the incidence of transitional cell bladder carcinoma and bladder cancer overall.

Although several investigations were conducted in Egypt dealing with the high rate of Schistosoma haematobium infection, data on other North African countries such as Libya are more limited. In a recent update from the Benghazi Cancer Registry, bladder cancer was the third most common malignancy in males accounting for 10 percent of the total number of cancer patients diagnosed from 2003 to 2005^7^.

In order to obtain more data on the nature of bladder tumours in Libya, we conducted a retrospective chart review of bladder cancer diagnoses from last 27 years (1983 to 2009), and collecting information on patient demographics, clinical presentation, tumour locations, and histopathological type.

## Methods

This study was a retrospective cohort analysis conducted among individuals who were diagnosed and/or treated for bladder cancer in Benghazi, using medical and pathological data from January 1st 1983 to December 31st 2009, a 27-year period. The data were collected from the bladder cancer register maintained in Hawari University Hospital, which is the main urological department in eastern part of Libya that provides diagnosis for the various conditions found in kidney, urethra, bladder, prostate, and testicular. The eastern part of Libya accounts for 28.5% (n = 1613749) of the country total population and consist of urban, suburban and rural areas. The Eastern region includes the following locations: Ajdabia, Beida, Benghazi, Derna, Kufra, Marj, Tobruk, and Wahat.

This study was approved by the Medical Research Committee of Hawari University Hospital. Informed patient consent was waived by the Research Committee due to the retrospective study design. All methods employed in this study were performed in accordance with the relevant guidelines. Age standardization of incidence rates was carried out by the direct method, using the world standard population^8^. The registry used statistical and data developed by the staff of the Modena Cancer Registry (MCR), Italy.

For assembling the study population, we combined the International Classification of Diseases, Ninth Revision, Clinical Modification (ICD-9-CM) codes for history of bladder cancer and included all consecutive patients who were diagnosed with bladder cancer during study period. Data on the age, sex, clinical presentation, imaging and histopathological features of bladder cancer were collected.

## Results

A total of 835 new cases of bladder cancer were diagnosed in Benghazi during the period of 1983 to 2009, of which 750 (89.8%) were male. The mean (standard deviation) age at diagnosis was 63.7 (14.7). Two-thirds of male patients (n = 594, 79.2%) were smokers while all female patients were non-smokers. Demographic and clinical characteristics of patients are demonstrated in Table 1.

**Table 1:** Demographic and clinical characteristics of patients.

Variables	Numbers (%)
**Age** (years), mean ± SD	63.7 (14.7)
**Gender**, *n* (%)	
Male	750 (89.8)
Female	85 (10.2%)
**Smoking status**	594 (79.2)
**Symptoms**, *n* (%)	
Frank hematuria	580 (69.5%)
Hematuria + I.V.S	100 (12%)
I.V.S	25 (3%)
Hematuria + O.V.S	96 (11.5%)
Others	34 (4%)
**Site**, *n* (%)	
Lateral Wall	522 (62.5%)
Base including BN	96 (11.5%)
Posterior Wall	58 (6.9%)
Anterior Wall	18 (2.1%)
Fundus	33 (4%)
Whole bladder	43 (5.1%)
Multifocal	65 (7.8%)
**Histopathology**, *n* (%)	
Transitional cell carcinoma	730 (87.4%)
Squamous cell carcinoma	74 (8.9%)
Undifferentiated	19 (2.3%)
Adenocarcinoma	10 (1.2%)
Non-secreting paraganglioma	1 (0.1%)
Mesonephric adenoma	1 (0.1%)

Abbreviations: BN, bladder neck; IVS, irritative voiding symptoms; n, number; OVS, obstructive voiding symptoms; SD, standard deviation.

Two-thirds of cases (n = 580, 69.5%) were presented with hematuria, while the most frequent histological types of bladder tumours were transitional cell carcinoma with 730 (87.4%) cases, and squamous cell carcinoma with 74 (8.9%) cases. The results of imaging studies and cystoscopy findings showed that two-thirds of bladder tumours (n = 522 cases, 62.5%) had lateral wall lesions. The majority of patients with bladder tumour (n = 696, 83.4%) had well differentiated tumours and only 139 patients (16.6%) had poorly differentiated tumours.

The age distribution and the age-specific incidence rates are presented in Table 2. The world age-standardized incidence rate was 13.1 and 1.9 per 100,000 for males and females, respectively. ASIRs of bladder cancer remained highest among men. This incidence rate increased with advancing age and reached the maximum in the age group above 70-years old (W-ASIR = 7.44).

**Table 2:** Crude and age-standardized incidence rate.

Age group	Bladder cancer		Age-specific incidence (per 10^5^ years)		World age- standardized incidence rate x 100,000 (W-ASIR)	
	Male	Female	Male	Female	Incidence Male	Incidence Female
10–19	6	0	0.13	0	0.03	0
20–29	25	0	0.58	0	0.10	0
30–39	23	3	0.63	0.09	0.10	0.01
40–49	68	9	3.63	0.50	0.44	0.06
50–59	148	5	13.82	0.44	1.66	0.05
60–69	255	9	37.22	1.47	3.35	0.13
70+	225	59	67.61	14.61	7.44	1.61
Total	750	85			13.12	1.86

## Discussion

The findings of this study revealed the over-all incidence of bladder cancer during 27 years was 13.1 and 1.9 per 100,000 for males and females, respectively. Majority of cases were male and the incidence increased via aging. Our finding is comparable with report from El Mistiri *et al*. (15.2 and 2.3 per 100,000 for males and females, respectively) that evaluated incidence of bladder cancer from 2003 to 2005^7^. However, the incidence in our study was lower than most developed countries (16.9 and 3.7 per 100,000 for males and females, respectively) but higher than the less developed countries (5.3 and 1.5 per 100,000 for males and females, respectively)^9^.

In the previous decade, studies have reported a decline in bladder cancer’s incidence and mortality in developed countries of Europe^10,11^ and Northern America^12^ through decreasing individuals exposure to tobacco smoke and occupational carcinogens such as aromatic amines which are the main risk factors for developing bladder cancer. On the other hand, the burden of cancer is expected to rise among developing countries, such as Libya^13^. This number is expected to rise by the increase in popularity of smoking among the Libyan population, and also by the shift to a lifestyle favouring obesity^14^, sedentary lifestyle, and diabetes mellitus^15^, all of which are directly linked to bladder cancer^16,17^. In addition, Libya was reported to have the fourth highest prevalence of Schistosoma haematobium infection in the Middle East and North African region, another risk factor directly associated with bladder cancer^18^.

In addition to the industrialization status, age and high life expectancy are other reasons to explain the high incidence of bladder cancer in developed countries. In accordance with previous reports^19^, the prevalence of bladder cancer in our study increased with aging, reaching peak numbers in patients between the ages of 50 to 79 years. Bladder cancer is a disease of the elderly and is rarely observed in individuals aged below 40 years. This is because with aging more time is provided for the neoplastic transformation, and the cumulative exposure to environmental carcinogens. Moreover, age related decrease in body detoxification and the decreased ability of older individuals to fully empty their bladder, which could also lead to their decreased water intake, are all among factors increasing the risk of bladder cancer in the elderly^20^. Hence, the prevalence of bladder cancer in Libya is expected to rise even more with an aging population in the foreseeable future.

In line with previous investigations, the incidence of bladder cancer in our study was much higher among males, two-third of whom were smokers. This difference could partly be explained by higher exposure of male gender to tobacco smoking and occupational hazards. The difference in hormonal levels and liver metabolism of bladder carcinogens are among other factors that were confirmed by human and animal studies to further increase the bladder cancer’s risk among males. In addition, in females, the estrogen inhibits the bladder carcinogenesis while the liver metabolism in male together with androgen promotes the bladder carcinogenesis^21^.

The histopathological types of bladder cancer were also analyzed in this study. In line with previous reports^22,23^, traditional cell carcinoma (also called urothelial carcinoma) accounted for the majority of cases. This was followed by squamous cell carcinoma, and adenoma carcinoma.

Previously, squamous cell carcinoma, which is associated to schistosomiasis infection, was responsible for most of the bladder cancer in African countries such as Egypt^5,24^. However, in the last decade there has been substantial drop in schistosomiasis associated bladder cancer and an increase in transitional cell carcinoma probably as a result of a smoking epidemic^5,25^. Nevertheless, the proportion of squamous cell carcinoma in our study was half of that reported by studies from Egypt^5,25^ where schistosomiasis infections are more common.

Public awareness about the nature and presentation of bladder cancer could aid in its early diagnosis and decrease its incidence and mortality. Encouraging increase in fluid intake^26^ as well as eating more vegetables and fruits^27,28^ could decrease the risk of bladder cancer. Consumption of broccoli and other cruciferous vegetables have significantly reduced the risk of bladder cancer in human and should be encouraged further^29^.

On the other hand, smoking^3,4^, exposure to occupational carcinogens^30^, obesity^17^, diabetes^15^ and schistosomiasis infections^31^ are among the main modifiable causative factors of bladder cancer, and hence should be addressed by public health to decrease the incidence of bladder tumours in the coming years.

Our study provided descriptive analysis of bladder cancer in eastern region of Libya, and calculated the overall world age-standardized incidence rate for period of 27 years. This study has a number of limitation. Due to limited available data, we could not measure the adjusted incidence rate for each individual year and only reported the overall incidence. In addition, we did not have enough information to identify the risk factors associated with the bladder cancer. Currently, there is lack of accurate and updated information on population size and health access in different Libyan locations. Having this information enable obtaining more detailed incidence rate and risk factor analysis for bladder cancer.

## Conclusions

In conclusion, the incidence of bladder cancer in Libya is lower than most developed countries, but higher than incidence from less developed countries. The risk of bladder cancer increases by aging and is more prevalent among males, the majority of whom were smokers. At the time where control of the main risk factors has resulted in a decline of bladder cancer’s incidence in developed countries, the incidence of this disease is expected to grow in developing countries such as Libya because of increase in smoking popularity, the shift to sedentary life, diabetes mellitus, and obesity.
